# A Comparison of Tooth Size and Arch Dimensions Among Measurements Taken Intraorally with 3D-Printed and Digital Models Obtained from Intraoral Scans

**DOI:** 10.4317/jced.61891

**Published:** 2024-08-01

**Authors:** Suthinee Kanokpoonsin, Supakit Peanchitlertkajorn, Nuntinee-Nanthavanich Saengfai, Supatchai Boonpratham

**Affiliations:** 1 Dr., D.D.S. Residency training in orthodontic department, Department of Orthodontics, Faculty of Dentistry, Mahidol University, Thailand; 2 Associate Professor, D.D.S., M.D.S. Diplomate, American board of Orthodontics, Diplomate, American Board of Dental Sleep Medicine, Diplomate, Thai Board of Orthodontics, Department of Orthodontics, Faculty of Dentistry, Mahidol University, Thailand; 3 Assistant Professor, D.D.S., M.Sc. Diplomate, Thai Board of Orthodontics, Department of Orthodontics, Faculty of Dentistry, Mahidol University, Thailand; 4 Associate Professor Dr., D.D.S., Ph.D. Diplomate, Thai board of Orthodontics, Department of Orthodontics, Faculty of Dentistry, Mahidol University, Thailand

## Abstract

**Background:**

To compare measurements of tooth size and arch dimensions among those taken directly intraorally with those made on digital and 3D printed models produced by intraoral scanning.

**Material and Methods:**

Sixty-six participants were recruited. Intraoral tooth size and arch measurements were taken intraorally with a digital caliper. Digital impressions were taken with an iTero® intraoral scanner. The three-dimensional digital models were measured using a 3D diagnostics tool (OrthoCAD software). The same digital models were used to fabricate physical models using a resin 3D printer (Elegoo Saturn). The measurements were repeated on 3D printed models by using the digital caliper. The recorded parameters included mesiodistal tooth widths, transverse, and antero-posterior dimensions. All measurements were repeated to assess intra- and inter- examiner reliability. The validity of each measurement method was assessed by repeated measures ANOVA with post-hoc pairwise comparisons (*p*<0.5).

**Results:**

The mean differences among three methods for all parameters were statistically significant (*p*<.05) but were considered to be clinically insignificant, except for the upper intercanine width. Direct intraoral measurements tend to be smaller than the digital and 3D printed models. The ICCs values indicated excellent intra- and inter-examiner reliability which demonstrates high reproducibility for all measurements on all model types.

**Conclusions:**

Direct intraoral measurements tend to be smaller than the digital and 3D printed models. However, the accuracy of measurements made directly intraorally, and on digital and 3D models from intraoral scans is clinically acceptable, except for the upper intercanine width.

** Key words:**Tooth measurements, Accuracy, Dental models, 3D printing, Digital model.

## Introduction

In dentistry, physical dental models hold a pivotal role within patient records, serving not only as diagnostic aids but also as imperative tools for evaluating treatment progression, outcomes, and facilitating inter-clinician communication (1,2).

Traditional methods of dental model fabrication, such as casting impressions in plaster or gypsum, are both effective and economical. However, the need for extensive space and careful long-term storage conditions to prevent deterioration presents a significant challenge for physical dental models. McGuinness *et al*. estimated that storing physical dental models for every 1000 patients requires up to 17 cubic meters of space, accompanied by substantial costs (3). Therefore, it is not surprising that clinicians would desire a more convenient and cost-effective means for records and storage while not compromising on their accuracy (4).

Since 1984, technological advancements have led to the era of digital dental workflows (5). One of the more recent innovations is the direct generation of three-dimensional (3D) models by intraoral scanning. Intraoral scanners are hand-held chairside devices that are able to directly generate 3D objects from multiple surface images. In short, a digital impression is produced, replacing the need for impression trays and materials which are often regarded as uncomforTable by the majority of patients (6,7). The shift towards digital dental impressions would eliminate the inherent problems related to physical model storage as mentioned above (3). Other potential beneﬁts include instant accessibility of 3D information, virtual treatment simulation, ease of digital file delivery to other clinicians or laboratories, and the ability to perform multiple diagnostic analyses (8).

Although, the use of physical dental models is still unavoidable in some circumstances, especially for the fabrication of dental appliances (2,9). The digital files from the intraoral scans can be exported in Standard Tessellation Language (STL) format to be 3D printed. Therefore, for digital impressions to effectively replace conventional techniques, evaluating the accuracy of digital models as well as their 3D-printed equivalents is of much importance.

While measurements on physical plaster models using vernier calipers are considered the gold standard due to their demonstrated accuracy and reliability (10-13), they are susceptible to errors stemming from various sources, including clinician skills, inaccurate impression tray dimensions, inappropriate amount of impression material and pouring technique, and distortions from disinfection procedures (14). To address these limitations, our study modified the gold standard of measurements to be taken directly from the patients’ dentitions using calipers instead of from plaster models. To our knowledge, this is the first study to use this approach on both maxillary and mandibular arches at the time of writing.

Hence, the purpose of this study was to compare tooth size and arch dimension measurements made on digital and 3D-printed models from intraoral scans with direct intraoral measurements taken manually using vernier calipers.

## Material and Methods

-Sample selection 

A total of 66 participants consisted of patients and staff who attended the Orthodontic Clinic at Mahidol University Dental Hospital, where they were initially screened and then recruited consecutively if they met the inclusion criteria. Written informed consent was obtained from all participants prior to the study. The inclusion criteria involved participants aged 12 or older, having fully erupted permanent dentition from the first molar to the contralateral first molar in the jaw, mild crowding (<4mm), no missing teeth, no prosthesis or extensive dental restorations more than three surfaces, absence of interproximal carious lesions or enamel defects affecting crown morphology, and no existing orthodontic appliances. Conversely, specific dental anomalies like hypodontia, supernumerary teeth, germination, fusion, concrescence, taurodontism, and patient with dentofacial deformities, were excluded from this study.

-Sample preparation

Detailed descriptions of the three measurement methods investigated in this study are given below.

1. Direct intraoral measurements

**Figure 1:**
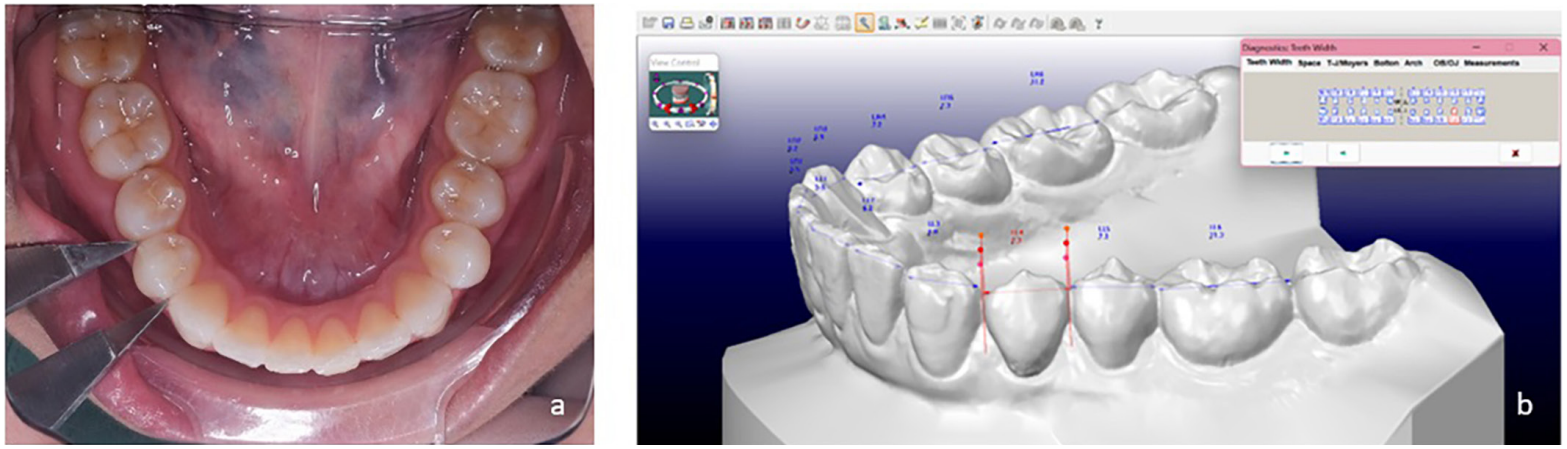
Representative images showing the measurement methods. a. Direct intraoral measurement of tooth width with Vernier calipers; b. Digital model measurement of tooth width with diagnostic tool.

One investigator (S.K) directly performed intraoral dental measurements using digital vernier calipers (Mitutoyo, Tokyo, Japan) which recorded up to two decimal places. The tips of the calipers were sharpened beforehand to allow accurate placement into the interproximal embrasures, and reset to the 0 value prior to measurement. The participants were asked to open their mouth comfortably and calipers were inserted from labial or buccal direction with the tips of the calipers parallel to occlusal plane of the tooth (Fig. 1a). The measurements were recorded up to two decimal places.

2. Digital models

Digital impressions of the participants’ dentitions were made with an intraoral scanner (iTero®, Align Technology, USA) by a single investigator (S.K) according to the manufacturer’s recommendations. The employed scanning strategy started with the occlusal surface as this has been reported to be more accurate than other scanning paths (15).

The scanned images were downloaded as STL files into a 3D software (OrthoCAD version 5.9, CADENT Inc, NJ, USA). A 14-inch computer screen with a resolution of 1920 x 1080 pixels and 64-bit color along with a standard computer mouse was used to manipulate the models and mark reference points. To allow proper visualization of each tooth, the software’s zoom, rotation, and panning features were utilized. Measurements on these digital dental models were then performed digitally using the diagnostics tool available in the same software, and we recorded up to two decimal places. Prior to each measurement of tooth width, the plane of measurement was adjusted to be parallel to the long axis of the tooth (Fig. 1b).

3. 3D-printed models

To create 3D-printed model, the STL files were downloaded, then prepared in a 3D software (Autodesk Meshmixer, version 3.5, Autodesk, Inc, CA, USA software), where all digital models were edited without the base and hollowed to a 2.5 mm wall thickness. These files were then exported into a 3D slicing software (Chitubox, version 1.8.1, CBD-Tech, Guangdong, China) to be placed in a horizontal orientation parallel to the printing platform, before being sent for 3D printing at a layer thickness of 100 microns in an LCD 3D printer (Saturn, ELEGOO Inc., Guangdong, China) with a resolution of 3840 x 2400. After printing process, the models were removed from the platform, washed in 99% isopropyl alcohol for 5 minutes and allowed to air dry before being post-cured in an ultraviolet chamber of 405 nm wavelength for 5 minutes (Mercury Plus, ELEGOO Inc., Guangdong, China). The measurements on these 3D-printed models were completed within 1 week after printing to reduce distortions from ageing (16). The same digital calipers (Mitutoyo, Tokyo, Japan) as in (1) above were used to obtain measurements of up to two decimal places.

-Data collection

The measurements taken for this study are described in  Table 1. The Federation Dentaire Internationale (FDI) system was used for tooth numbering.

**Figure 2:**
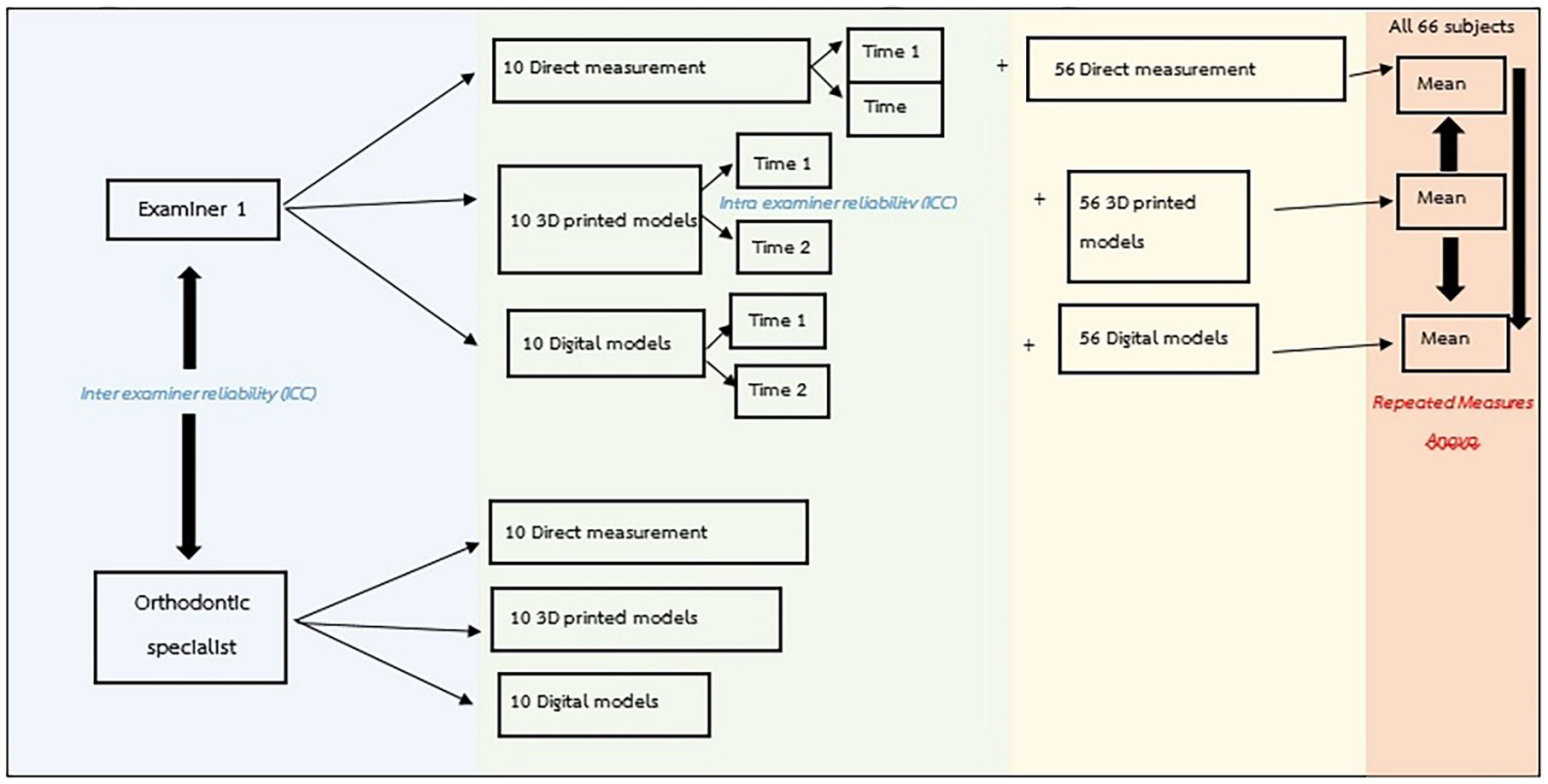
Schematic illustration of the measurement work flow for the two investigators of the study.

The investigators for this study consisted of one orthodontic resident (S.K.) and one orthodontic specialist (S.B.) from the Department of Orthodontics, Mahidol University as the primary and secondary investigators respectively. All investigators were blinded to the identity of the models by assigning a new random number for each set of measurements. The primary investigator (S.K.) performed all measurements twice for 10 participants to evaluate intra-examiner reliability, while the secondary examiner (S.B.) also made all measurements in order to evaluate inter-examiner reliability. In order to ascertain the reliability of the first investigator, both scores should be greater than 0.9. Subsequently, the primary investigator (S.K.) completed all measurements in the remaining 56 participants, and the results from the total of 66 participants measured by the primary investigator were analyzed and reported (Fig. 2).

-Statistical analysis

Statistical analyses were performed using the Statistical Package for Social Sciences software (SPSS, version 21.0, IBM, Chicago, IL, USA). Intraclass correlation coefficients (ICCs) were calculated to determine inter- and intra-examiner reliability. ICC values of between 0.75 to 0.9 would indicate good reliability, and above 0.9 would be excellent reliability (17). Repeated measures ANOVA was run to compare the validity among the three measurement methods, followed by post-hoc using Bonferroni tests for pairwise comparisons. The level of statistical significance was set at a *p-value* of <0.05. For each outcome, mean differences of 0.3 mm and above for tooth width and 0.4 mm or greater for transverse and anteroposterior parameters were considered to be clinically relevant (8,18-20).

## Results

The total group comprised 66 participants, 49 females and 17 males, with a mean age of 21.7 years. ICC values indicated excellent inter- and intra-examiner reliability which demonstrates high reproducibility for all measurements on all model types. (ICCs values exceeded 0.90).

Tooth-width (mesiodistal) measurements

The means and standard deviations for tooth width measurements for each method are shown in  Table 2, while the pairwise comparisons including mean differences are shown in  Table 3.

Statistically significant differences (*p*<.05) were found in approximately two thirds of the measured teeth, with discrepancies being more commonly found among posterior teeth compared to anterior.

The results of the pairwise comparisons ( Table 3) showed that the direct measurements were statistically significant, on average, 0.03 mm and 0.05 mm less than those measured on the 3D printed and digital models, respectively, while measurements from the 3D printed models were statistically significant on average 0.01 mm smaller than from the digital models.

The largest mean differences of 0.22 ± 0.4 mm were found at the mandibular right first molar (tooth 46), comparing between the direct method with both 3D printed and digital models, and these differences were statistically significant (*p*<.05). On the other hand, the smallest mean differences of 0.00 mm were recorded at the same tooth but comparing between 3D printed and digital models. For all pairwise comparisons, no mean differences exceeded 0.3 mm, indicating that the discrepancies between the three methods were not clinically significant.

Transverse measurements

For transverse measurements ( Table 4), all dimensions showed statistically significant differences between the three methods, except for the upper intermolar width (*p*>.05)

The pairwise comparisons ( Table 5) showed that transverse arch dimensions measured using the direct method were, on average, 0.13 mm and 0.27 mm smaller than those measured on 3D printed and digital models, respectively, while transverse measurements made on 3D printed models were 0.14 mm smaller than on digital models. These differences were also statistically significant (*p*<.05).

The largest mean differences were consistently observed for upper intercanine width, particularly mean difference between direct and digital methods of 0.62 mm. As this was greater than 0.4 mm, this result was considered to be clinically significant. All other transverse measurements showed mean differences below the clinical threshold of 0.4 mm.

Anteroposterior measurements

For measurements of arch length ( Table 6, Table 7), statistically significant differences between methods were found in the lower left arch length dimensions (*p*<.05). Significant differences were present between the direct method and 3D printed models, as well as between the 3D printed and digital models. These two pairs also displayed the greatest mean differences among all pairwise comparisons of 0.13 and 0.14 mm respectively, but as they did not exceed 0.40 mm, it would not be considered clinically significant.

On average, measurements with the direct method were 0.09 mm and 0.02 mm greater than those taken from 3D printed and digital models, respectively, but the difference in the latter was not statistically significant. Conversely, 3D printed models tended to yield measurements that were significantly less by an average of 0.07 mm than those made digitally.

## Discussion

In any analysis involving landmark identification, experience and calibration are necessary to improve the accuracy and reliability of the measurements (21). Before formal data collection, the investigators in this study were trained and calibrated for all measurements, and a high level of excellent reliability was obtained.

In this investigation, the direct method, 3D printed models, and digital models yielded measurements ranging from lowest to highest for mesiodistal and transverse dimensions, respectively. Cuperus *et al*. explained that the the difficulties in scanning contact points or interproximal regions in the dental arch leads to small amounts of artifacts or gaps in these areas, which is ‘filled in’ by artificial intelligence technology and this can result in slight variation in contact point location, meaning that measurement taken from digital models tend to be greater than those from 3D printed model (22). Additionally, due to the nature of the 3D printing resins, 3D printed models commonly undergo polymerization shrinkage of about 10% (23). These limitations in the digital workflow should be of note to clinicians as it would influence the accuracy of measurements taken from digital scans and 3D printed models.

Looking more closely at the mesiodistal tooth measurements, the largest mean difference was seen at the mandibular right first molar when comparing the direct method with 3D-printed models and digital models. Conversely, the smallest mean difference was found between 3D-printed and digital models at the same tooth. These results suggest that it may be more difficult to measure teeth directly in the mouth than extraorally on tooth models, particularly for the lower right posterior teeth. A similar finding was reported in the study by Hunter and Priest who compared measurements made directly intraorally with those on plaster models (24). They observed that the measurement of posterior teeth was also more challenging due to problems with access. Nevertheless, these difficulties were not encountered for the anterior teeth.

For transverse measurements, the upper intercanine width consistently produced the largest mean differences for all three methods. Particularly, the direct method revealed a clinically significant shorter distance compared to the digital method. These findings were supported by the results of prior study, which also indicated significantly shorter distance in upper intercanine width when measurements were taken directly intraorally compared to those derived from digital model (25). However, there could be several reasons for this discrepancy. Initially, within our participant sample, we commonly encountered canine cusp tips which were worn due to attrition, and this would have complicated the landmark identification, leading to more measurement errors as observed by Camaedella *et al*. (26). Secondly, as comparisons with the digital models produced more significant discrepancies, we hypothesize that the two-dimensional digital models could be even more challenging to use in identifying certain anatomical tooth landmarks, especially cusp tips. In these cases, tactile sensation made directly intraorally or on physical models could improve the localization of such anatomical landmarks. The use of digital models when locating landmarks involving pits and grooves, however, did not seem to pose this difficulty. This was demonstrated by the upper intermolar width which was the single transverse measurement that did not reach statistical significance in any pairwise comparisons between methods, as the landmarks utilized the central fossae and not cusp tips. Therefore, the accuracy of our results could have been improved by ruling out participants with evident tooth wear that would complicate the identification of certain anatomical tooth landmarks.

For anteroposterior measurement, the mean differences were mostly statistically insignificant. All mean differences ranged from 0 to 0.14 mm, which was well below the clinical threshold of 0.4 mm.

As the 3D printed model measurements tend to always be smaller than digital models, the problem of the polymerization shrinkage could again be the cause for this. Even so, the shrinkage of 3D printed models does not seem to occur in a uniform fashion, and this could be due to a whole range of factors related to the 3D printing process such as post-curing, washing, print orientation, layer thickness, 3D printer type, and 3D printing material (27-29). Moreover, the dimensional stability of 3D-printed dental models is only reliable up to 3 to 4 weeks after printing, after which it tends to deteriorate (16). In order to minimize errors, we completed all measurements from the 3D-printed models within a week after printing. Future studies could assess the proper parameters related to the 3D printing process to further improve their accuracy.

Within the limitations of our study, it is evident that each measurement method has its strengths and drawbacks. While both digital and 3D-printed dental models have no problems with physical access, the former method may be subject to overestimation. The 3D-printed models as mentioned previously are not free from fabrication and time-dependent errors either. Thus, it is in our opinion that direct measurements have the advantages of being cost-effective and time-efficient as only a pair of calipers is needed to obtain results immediately chairside. Our results have also shown that measurements made directly intraorally are consistently reliable for the anterior teeth, and this is sufficient for management of anterior aesthetics by the various branches of dentistry.

Nevertheless, with this knowledge in mind, clinicians can select the method which best suits their practice to aid in diagnosis and treatment planning. For whichever measurement method, a learning curve which can be assisted by proper training and calibration is necessary to improve the accuracy and reliability of measurements.

## Conclusions

Direct intraoral measurements tend to be smaller than measurements made on digital and 3D printed models, especially for mesiodistal tooth widths and transverse measurements. However, the magnitude of the differences does not appear to be clinically relevant, except for the upper intercanine width, which is complicated by challenges in identifying anatomical landmarks, particularly in the region affected by canine attrition.

## Figures and Tables

**Table 1:** Definitions of each measurement taken for this study.

Mesiodistal Tooth Width	Greatest mesiodistal diameter from the anatomic mesial contact point to the anatomic distal contact point in each tooth, parallel to the occlusal surface.
Upper Intercanine width	Distance between the cusp tip of the upper left canine to cusp tip of the upper right canine
Lower Intercanine width	Distance between the cusp tip of the lower left mandibular canine to cusp tip of the lower right canine
Upper Intermolar width	Distance between the central fossa of the upper left 1st molar to the central fossa of the upper right 1st molar
Lower Intermolar width	Distance between the tip of the distobuccal cusp of the lower left 1st molar to the tip of the distobuccal cusp of the lower right 1st molar
Arch length	Distance from contact point of central incisor to mesial contact point of 1^st^ molar

**Table 2:** Tooth width measurements according to method.

Tooth	Direct		3D-Printed		Digital		df	F	P value
	Mean (mm)	SD (mm)	Mean (mm)	SD (mm)	Mean (mm)	SD (mm)			
11	8.68	0.57	8.67	0.57	8.65	0.57	2	2.386	0.1
12	7.22	0.50	7.21	0.50	7.19	0.48	2	1.575	0.215
13	8.10	0.48	8.10	0.48	8.08	0.48	2	3.966	0.024*
14	7.65	0.46	7.60	0.44	7.64	0.44	2	5.683	0.005*
15	7.12	0.47	7.11	0.47	7.16	0.46	2	6.286	0.002*
16	10.07	0.56	10.16	0.53	10.29	0.49	2	16.092	<0.001*
21	8.71	0.55	8.69	0.53	8.69	0.53	2	0.834	0.439
22	7.23	0.53	7.19	0.53	7.17	0.52	2	7.931	0.001*
23	8.03	0.49	8.03	0.50	8.05	0.48	2	1.597	0.210
24	7.64	0.46	7.65	0.42	7.65	0.42	2	0.114	0.892
25	7.14	0.46	7.08	0.45	7.16	0.42	2	5.102	0.009*
26	9.98	0.60	10.17	0.55	10.19	0.47	2	33.312	<0.001*
31	5.47	0.39	5.51	0.37	5.53	0.38	2	9.098	<0.001*
32	6.08	0.43	6.12	0.42	6.10	0.43	2	1.822	0.170
33	6.97	0.40	7.04	0.40	7.00	0.41	2	12.405	<0.001*
34	7.52	0.44	7.56	0.44	7.55	0.42	2	2.898	0.062
35	7.51	0.42	7.46	0.42	7.50	0.42	2	4.992	0.010*
36	11.47	0.51	11.46	0.51	11.45	0.48	2	0.220	0.803
41	5.43	0.38	5.50	0.38	5.51	0.40	2	15.955	<0.001*
42	6.05	0.42	6.13	0.53	6.07	0.43	2	1.363	0.263
43	6.99	0.47	7.03	0.48	6.99	0.47	2	3.329	0.042*
44	7.47	0.41	7.53	0.41	7.53	0.41	2	3.488	0.037*
45	7.29	0.42	7.38	0.41	7.40	0.40	2	8.642	<0.001*
46	11.15	0.57	11.37	0.56	11.37	0.51	2	10.318	<0.001*

*Statistically significant at *p*<.05.

**Table 3:** Pairwise comparisons between methods for measurements of tooth width.

Tooth	Direct vs. 3D Printed			Direct vs. Digital			3D Printed vs. Digital		
	Mean Difference (mm)	SD (mm)	P value	Mean Difference (mm)	SD (mm)	P value	Mean Difference (mm)	SD (mm)	P value
11	0.01	0.09	0.763	0.03	0.12	0.096	0.02	0.12	0.433
12	0.01	0.18	1.000	0.03	0.13	0.253	0.02	0.18	1.000
13	0.00	0.15	1.000	0.02	0.12	0.359	0.02	0.09	0.053
14	0.05	0.14	0.006*	0.01	0.11	1.000	-0.04	0.12	0.011*
15	0.01	0.13	1.000	-0.04	0.14	0.040*	-0.05	0.12	0.003*
16	-0.09	0.36	0.125	-0.22	0.37	0.000*	-0.13	0.25	<0.001*
21	0.02	0.13	0.709	0.02	0.15	0.665	0.00	0.10	1.000
22	0.04	0.11	0.027*	0.06	0.11	<0.001*	0.02	0.13	0.736
23	0.00	0.20	1.000	-0.02	0.13	0.449	-0.02	0.16	0.755
24	-0.01	0.17	1.000	-0.01	0.16	1.000	0.00	0.15	1.000
25	0.06	0.27	0.269	-0.02	0.20	0.966	-0.08	0.21	0.007*
26	-0.19	0.38	0.001*	-0.21	0.43	<0.001*	-0.02	0.20	0.625
31	-0.04	0.12	0.038*	-0.06	0.10	<0.001*	-0.02	0.13	0.482
32	-0.04	0.21	0.398	-0.02	0.22	1.000	0.02	0.12	0.445
33	-0.07	0.13	<0.001*	-0.03	0.12	0.071	0.04	0.12	0.021*
34	-0.04	0.18	0.274	-0.03	0.11	0.068	0.01	0.15	1.000
35	0.05	0.17	0.028*	0.01	0.16	0.890	-0.04	0.11	0.028*
36	0.01	0.22	1.000	0.02	0.24	1.000	0.01	0.17	1.000
41	-0.07	0.14	<0.001*	-0.08	0.13	<0.001*	-0.01	0.13	1.000
42	-0.08	0.39	0.364	-0.02	0.17	1.000	0.06	0.40	0.720
43	-0.04	0.16	0.118	0.00	0.13	1.000	0.04	0.12	0.051
44	-0.06	0.17	0.030*	-0.05	0.19	0.087	0.00	0.11	1.000
45	-0.09	0.25	0.010*	-0.11	0.22	<0.001*	-0.02	0.16	0.775
46	-0.22	0.4	<0.001*	-0.22	0.39	<0.001*	0.00	0.20	1.000
Overall	-0.03	0.06	0.0001*	-0.05	0.06	<0.0001*	-0.01	0.05	0.030*
Limits of Agreement	-0.15,0.08			-0.15,0.06			-0.11,0.09		

*Statistically significant at *p*<.05

**Table 4:** Transverse measurements according to method.

Transverse Dimension	Direct		3D-Printed		Digital		df	F	P value
	Mean (mm)	SD (mm)	Mean (mm)	SD (mm)	Mean (mm)	SD (mm)			
UICW	35.23	2.06	35.54	2.01	35.85	2.13	2	57.360	<0.001*
LICW	27.49	2.10	27.45	2.03	27.75	2.07	2	64.000	<0.001*
UIMW	48.27	2.43	48.39	2.52	48.32	2.38	2	1.656	0.195
LIMW	48.85	2.78	48.97	2.80	48.98	2.80	2	3.264	0.041*

UICW: Upper intercanine width; LICW: Lower intercanine width; UIMW: Upper intermolar width; LIMW: Lower intermolar width *Statistically significant at *p*<.05

**Table 5:** Pairwise comparisons between methods for measurements in the transverse dimension.

Transverse Dimension	Direct vs. 3D Printed			Direct vs. Digital			3D Printed vs. Digital		
	Mean Difference (mm)	SD (mm)	P value	Mean Difference (mm)	SD (mm)	P value	Mean Difference (mm)	SD (mm)	P value
UICW	-0.31	0.45	<0.001*	-0.62	0.53	<0.001*	-0.31	0.43	<0.001*
LICW	0.04	0.50	1.000	-0.26	0.58	0.002*	-0.30	0.41	<0.001*
UIMW	-0.12	0.58	0.280	-0.05	0.49	1.000	0.07	0.56	0.976
LIMW	-0.12	0.49	0.183	-0.13	0.41	0.041*	-0.01	0.44	1.000
Overall Transverse	-0.13	0.26	0.0002*	-0.27	0.28	<0.0001*	-0.14	0.26	0.0001*
Limits of Agreement	-0.63,0.38			-0.82,0.29			-0.65,0.37		

UICW: Upper intercanine width; LICW: Lower intercanine width; UIMW: Upper intermolar width; LIMW: Lower intermolar width *Statistically significant at *p*<.05

**Table 6:** Anteroposterior measurements according to method.

Anteroposterior Dimension	Direct		3D-Printed		Digital		df	F	P value
	Mean (mm)	SD (mm)	Mean (mm)	SD (mm)	Mean (mm)	SD (mm)			
Upper right arch length	35.89	2.10	35.78	1.96	35.90	1.95	2	4.788	0.012*
Upper left arch length	35.97	2.17	35.89	2.15	35.89	2.06	2	2.853	0.065
Lower left arch length	30.67	1.76	30.54	1.74	30.68	1.73	2	8.925	<0.001*
Lower right arch length	30.32	1.87	30.29	1.84	30.32	1.85	2	0.341	0.712

*Statistically significant at *p*<.05

**Table 7:** Pairwise comparisons between methods for measurements in the anteroposterior dimension.

Anteroposterior Dimension	Direct vs. 3D Printed			Direct vs. Digital			3D Printed vs. Digital		
	Mean Difference (mm)	SD (mm)	P value	Mean Difference (mm)	SD (mm)	P value	Mean Difference (mm)	SD (mm)	P value
Upper right arch length	0.11	0.39	0.065	-0.01	0.54	1.000	-0.12	0.42	0.076
Upper left arch length	0.08	0.27	0.071	0.08	0.42	0.415	0.00	0.40	1.000
Lower left arch length	0.13	0.35	0.009*	-0.01	0.4	1.000	-0.14	0.31	0.002*
Lower right arch length	0.03	0.33	1.000	0.00	0.40	1.000	-0.03	0.35	1.000
Overall Anteroposterior	0.09	0.21	0.0012*	0.02	0.28	0.6125	-0.07	0.25	0.0214*
Limits of Agreement	-0.33-0.51			-0.53-0.57			-0.55-0.41		

*Statistically significant at *p*<.05

## Data Availability

The datasets used and/or analyzed during the current study are available from the corresponding author.
